# Application of team-based learning to ophthalmology in China

**DOI:** 10.3389/fpubh.2022.922325

**Published:** 2022-10-10

**Authors:** Wenyi Wu, Li Pu, Endong Zhang, Siqi Xiong, Xiaolai Zhou, Xiaobo Xia, Dan Wen

**Affiliations:** ^1^Department of Ophthalmology, Xiangya Hospital, Central South University, Changsha, China; ^2^Hunan Key Laboratory of Ophthalmology, Changsha, China; ^3^National Clinical Research Center for Geriatric Disorders, Xiangya Hospital, Central South University, Changsha, China; ^4^State Key Laboratory of Ophthalmology, Zhongshan Ophthalmic Center, Sun Yat-sen University, Guangzhou, China

**Keywords:** TBL, ophthalmology, efficiency, satisfaction, application

## Abstract

**Objectives:**

The purpose of this study was to explore whether team-based learning (TBL) was more effective than traditional didactic lectures (TDLs) in improving medical students' problem-solving and study skills in the clinical course of ophthalmology. In addition, we were also concerned about Chinese students' satisfaction with TBL.

**Methods:**

Our study program involved 275 students of the 5-year clinical medicine program from Central South China University, of which 140 were enrolled in a modified TBL course. A questionnaire that included closed-ended and open-ended items was distributed to students immediately following the completion of the TBL session, and 108 valid questionnaires were collected. Descriptive statistics were used to analyze quantitative data. The effects of the TBL module on students' performance were measured between the groups using a one-way between-group analysis of variance (ANOVA) test by the individual readiness assurance test (IRAT), the group readiness assurance test (GRAT), and final examination scores (FESs), compared with a class without the TBL session.

**Results:**

With our modified TBL strategy, 140 students achieved a mean test score of 72.65 on test questions that assessed their knowledge of ophthalmology compared to 135 students who achieved a mean score of 70.8 using the TDL method (*p* = 0.3434). The performance in a pre-class quiz was significantly better in the GRAT compared to the IRAT. In comparison to the TDL session, the modified TBL was preferred and acceptable by most medical students.

**Conclusions:**

By applying the modified TBL to ophthalmology, students improved their performance, self-study, and teamwork, and their class engagement and satisfaction were enhanced. However, TBL should be further optimized and developed to enhance educational outcomes.

## Introduction

Team-based learning (TBL) was first popularized at the University of Oklahoma in the 1970's due to increasing class sizes and concerns about its effectiveness (1, 2). It is an educational strategy characterized by individual work, teamwork, and immediate feedback, enabling students to discuss and follow a structured process to enhance their active engagement (3). As there was an increase in the number of medical students, TBL received much attention, especially in large-group teaching (4, 5), and it was widely adopted in medical pedagogical approaches across the world (6–8). Moreover, with the explosion of knowledge and population, it is more important for students to collaborate in this involution area, which is the focus of many training in TBL pedagogy (9).

However, the most popular education method in China is the traditional didactic lecture (TDL) (10). These students prefer to follow what teachers talked about active participation in a class discussion. The major shortcoming of TDL is that students receive information passively and have little opportunity to express an opinion and exercise critical thinking. They are reluctant to spend much time on the proactive preview of textbooks and have little interest in active thinking; thus, most students cannot apply the knowledge flexibly. Thus, the introduction of an active learning approach, such as TBL, in education attracts attention (11, 12). More importantly, TBL performed better compared with TDL (13) and permitted a large student–teacher ratio, which greatly fits the status of Chinese medical education without teachers and classrooms (14, 15).

To improve the medical teaching method in large classes, we decided to apply TBL an ophthalmology clerkship and evaluate its efficiency and student's learning abilities. According to a previous study, we launched the modified TBL with the help of advanced technology of Superstar app and lecture, which intends to attract the students' attention and interest. The lecture in the TBL covers clinical development or biological advances in each unit. At the end of our pedagogical method, we assessed TBL satisfaction and efficiency using a questionnaire. In addition, we investigated whether any other improvement of TBL would benefit our students, based on the feedback from students. In conclusion, these findings can provide insights into an understanding of the ways of effective teaching and some TBL problems in medical education, which may also be referable to other countries, especially those countries with similar pedagogical structures as China.

## Methods

### Participants

Our study population comprised third year medical students (clinical medicine) from the Central South China University. In the third year, the students moved to the hospital, where they were rolled into various medical subspecialties including ophthalmology, and underwent clinical clerkship during our TBL course. Approximately 80 faculty members from the ophthalmology department were part of the TBL teacher. In total, 140 students participated in the TBL session. The Human Research Ethics Committee of Central South University approved this study. All methods were carried out in accordance with relevant guidelines and regulations.

### TBL learning outcomes

The score of the individual readiness assurance test (IRAT) and the group readiness assurance test (GRAT), including the score of a comprehensive clinical application at the end of the class, and the final examination scores (FESs) were selected to provide learning outcomes in our assessment. Furthermore, our results also include satisfaction questionnaires and the evaluation of teaching goals.

### Structure of TBL

The duration of the TBL session was 150 min with two breaks. The session was held outside the students' regular weekly schedule to preview the MOOC video and book. The students were randomly divided into 10 teams per class and evenly distributed by gender, with each team consisting of ~14 members. Two or three teachers were randomly allocated to these groups to participate in the discussion section in the GRAT and the comprehensive clinical application task.

### TBL process

All students were allocated required compulsory readings for pre-class reading and pre-recorded lectures to review.

### Individual readiness assurance test

At the beginning of the class, all students were required to complete an online quiz with the Superstar app. The quiz consisted of 10 multiple-choice questions, with a single best answer or multi-choice for each question. Questions were aligned with pre-class reading and pre-recorded lectures. Students were provided with a 15-min window to complete the quiz and were provided with their total score upon completion.

### Group readiness assurance test

The same IRAT quiz was repeated by the other students in their teams. The test was conducted online, and students submitted one answer per team, with the intention of promoting a discussion to establish a team consensus.

### Immediate feedback and clarification from facilitators

Then, the correct answers were released and explained, giving immediate feedback on the team's responses in ~30 min. The teacher offered clarification, particularly when individuals or teams had problems.

### Extended lecture

Our teachers gave an interesting lecture with the intention of exploiting the horizon of medical students in learning ophthalmology and the content of the lecture which includes the progress of scientific research, the operation of each disease, and advanced medical technologies. This lecture lasted for about 1 h. We added this section with the intention of increasing students' interest in ophthalmology.

### Clinical problem-solving activities

**Figure 1:**
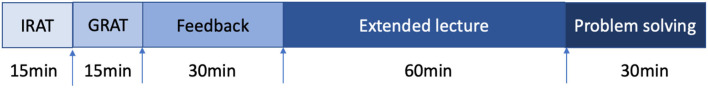
Time schedule of team-based learning (TBL).

The students worked in their teams on problem-solving activities, using the knowledge consolidated through the prior steps. In the immediate feedback session, there was an opportunity for students to initiate a discussion and challenge the answers. The overall plan is shown in Figure 1.

### TBL modification

Each TBL class comprises a professional team of facilitators and three ophthalmologists. These facilitators received prior training in TBL facilitation by either attending a face-to-face training semester. In our modification, we provided additional professional research lectures and specialty clinical lectures in the international language, which include elements such as how to prompt clinical reasoning through questions.

### Data collection

We collected data using multiple questionnaires regarding TBL experiences and study outcomes. The questionnaire was distributed to the student immediately following the completion of the TBL session. These questionnaires included closed items (using a five-point Likert scale, with 1 being “strongly disagree,” and 5 being “strongly agree”).

The interviewers used a guide that contains seven standardized, open-ended interview questions about general impressions of the dissemination of TBL in the school, the degree of use of TBL in specific courses, scholarship on TBL, and the future. A series of prompts were included to ensure that each question was explored in similar detail between interviewers. A copy of the interview guide is provided in Table 1. All interviews were conducted over the telephone.

**Table 1:** Questionnaire results about the teaching objectives among team-based learning (TBL) students.

**Question**		**Frequency %**
**TBL can meet the teaching requirements of the syllabus**		
Strong agree	13	12.04%
Agree	37	34.26%
Neutral	22	20.37%
Disagree	31	28.7%
Strong disagree	5	4.63%
**Have you grasped the key points and difficulties in TBL class?**		
Strong agree	9	8.33%
Agree	54	50%
Neutral	17	15.74%
Disagree	20	18.52%
Strong disagree	8	7.41%
**TBL teaching content has a wide coverage**		
Strong agree	18	16.67%
Agree	35	32.41%
Neutral	39	36.11%
Disagree	12	11.11%
Strong disagree	4	3.7%
**TBL makes us more efficient in achieving our goals and learning knowledge**		
Strong agree	8	7.41%
Agree	18	16.67%
Neutral	45	41.67%
Disagree	28	25.93%
Strong disagree	9	8.33%
**whether the knowledge in the preview has been strengthened in the discussion**		
Strong agree	21	19.44%
Partially agree	67	62.04%
Disagree	20	18.52%
**Clinical problem-solving is helpful to clinical thinking and the application of knowledge**		
Strong agree	30	27.78%
Partially agree	66	61.11%
Disagree	12	11.11%
**Team discussion deepens the understanding of knowledge**		
Strong agree	21	19.44%
Partially agree	63	58.33%
Disagree	24	22.22%
**We will be spent more time on pre-class preparation**		
Strong agree	40	37.04%
Partially agree	62	57.41%
Disagree	6	5.56%
**IRAT at the beginning of TBL is not difficult**		
Strong agree	44	40.74%
Partially agree	59	54.63%
Disagree	5	4.63%

### Data analysis

We used the descriptive statistics method to analyze our questionnaire data. Thematic analysis was used to build an understanding of the students' experience of the TBL session. A portion of the data was read by the first author and analyzed to identify initial themes. Following the negotiation of meaning with the second author, a coding framework was developed and applied to the full data set.

Differences in proportions between the TBL and PL groups were tested using the χ^2^ test; differences in the mean of the IRAT and GRAT scores were tested using a two-sample *t*-test if the assumptions of normality and homogeneity were satisfied; otherwise, the nonparametric Mann–Whitney *U*-test was applied. A one-way analysis of variance (ANOVA) were used to compare the IRAT, GRAT and final exam scores among students stratified according to BOLs. All analyses were performed using SPSS software version 22.0 (SPSS, Inc., Chicago, IL, USA).

### Ethics approval

The Institutional Review Board (IRB) of Xiangya Hospital, Central South University approved this study. Participation was voluntary, consent forms were signed, and anonymity was guaranteed. All methods were carried out in accordance with relevant guidelines and regulations.

## Results

A total of 140 students participated in our TBL method (Table 1). Of these, 108 questionnaires were collected at the end of our class. The mean age was 20.8 ± 0.69 years [all values were expressed as mean ± standard error of the mean (SEM)]; 43.52% were men and 64.81% resided in the city.

### Class performance

**Figure 2:**
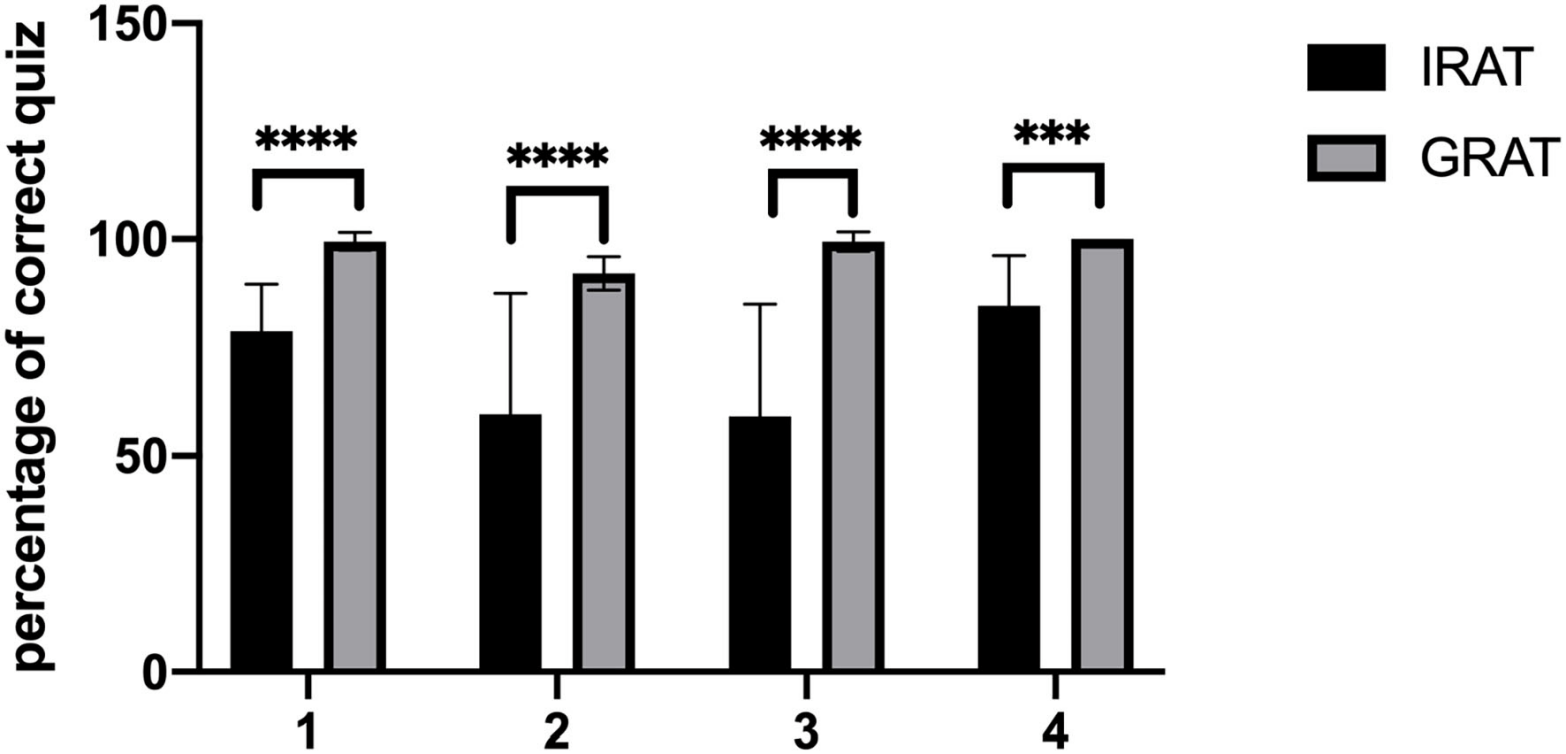
The score of the group readiness assurance test (GRAT) and the individual readiness assurance test (IRAT). ***P < 0.001, ****P < 0.0001 between the two compared groups by unpaired *t* test.

**Figure 3:**
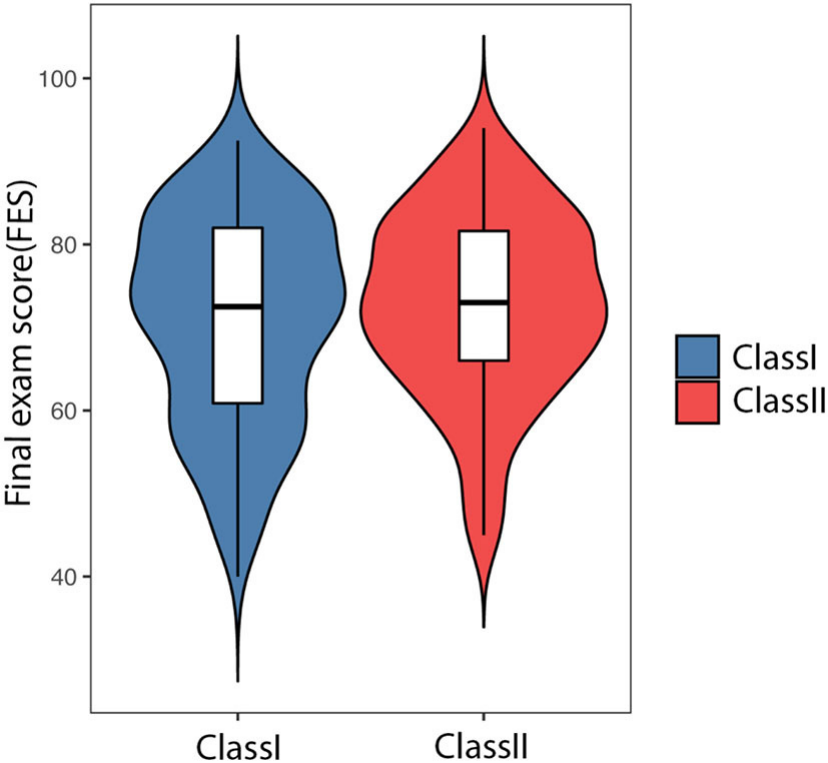
The final exam score of traditional didactic lecture (TDL) and TBL education. Class I using TDL and Class II using TBL.

The proportion of correct answers was significantly higher in the GRAT than in the IRAT (Figure 2) and with more practice with TBL training, and the performance of the IRAT gets better and better each time (Figure 2). These results confirmed that problem-solving was more effective in groups than in individuals. The average score of the final exam (Figure 3) with TBL was 72.65 while the class without TBL training was 70.8. There was no significant difference between the students with or without TBL (*p* = 0.3434). However, the number of failed students is much higher in TDL than in TBL (31 vs. 17) and the number of students passed is more in TBL than in TDL (36 vs. 25). Fair, good, and excellent students are similar in number in both TBL and TDL (43 vs. 40, 37 vs. 35, and 7 vs. 5).

The data showed that TBL training could at least match our previous traditional method, and some other capabilities, such as collaboration and expression, demonstrated strength in this educational method.

### Evaluation of TBL on teaching objectives

At the end of the module, students were asked to rate their experience with TBL. We first evaluated the teaching objectives of the TBL model, and the result is shown in Table 1. Approximately 33.33% disagreed that TBL could meet the teaching syllabus, and the percentage became smaller as ~25.93% disagreed that they grasped the key point and the difficulties in the TBL class. Approximately 14.81% of the students disagreed that the teaching content extended more widely in TBL than in TDL. Regardless of the efficiency in learning knowledge with TBL training only, 24.08% of the students agree that they are enhanced than before. Next, from the evaluation of the part of the discussion and clinical problem-solving in TBL, we obtained a higher percentage of agreeing with their role in learning knowledge. Furthermore, about 94.45% of the students are willing to spend more time to preview. This is an important self-learning skill and increases their competitiveness in future studies.

### Evaluation of learning skills and interest in ophthalmology

We next investigated the effects on learning ability and study interest, with a focus on oral expression, independent thinking, and time management, with 20.37, 18.52, and 25.93% disagreeing, respectively (Table 2); there seems no significant difference between these skills. Some other learning skills, such as self-learning and collaboration, are thought to be improved at percentages of 77.78% and 64.81%. We continued to assess the interest in ophthalmology and the time spent to include the expansion of the theme's scope of knowledge, and the result shows that students agree or partially agree with this effect of TBL in 64.81, 69.44, and 92.59%.

**Table 2:** Learning ability and interest.

**Question**		**Frequency %**
**TBL helps to improve our ability to expression**		
Strong agree	8	7.41%
Agree	20	18.52%
Neutral	53	53.7%
Disagree	18	16.67%
Strong disagree	4	3.7%
**TBL has trained our independent thinking ability**		
Strong agree	8	7.41%
Agree	36	33.33%
Neutral	44	40.74%
Disagree	17	15.74%
Strong disagree	3	2.78%
**More effective utilize and controllable of time to learning in TBL**		
Strong agree	7	6.48%
Agree	16	14.81%
Neutral	49	45.37%
Disagree	29	26.85%
Strong disagree	7	6.48%
**TBL improves our ability to self-learning**		
Strong agree	28	25.93%
Partially agree	56	51.85%
Disagree	24	22.22%
**TBL improves team collaboration ability**		
Strong agree	23	21.3%
Partially agree	62	57.41%
Disagree	23	21.3%
**TBL stimulates our interest in ophthalmology or clinical medicine**		
Strong agree	14	12.96%
Partially agree	56	51.85%
Disagree	38	35.19%
**TBL makes us more willing to spend time on ophthalmology learning**		
Strong agree	16	14.81%
Partially agree	59	54.63%
Disagree	33	30.56%
**TBL extends the knowledge of ophthalmology with extra-curricular content**		
Strong agree	51	47.22%
Partially agree	49	45.37%
Disagree	8	7.41%

### Evaluation of team working skills and clinical ability

The aim of TBL was to increase the collaboration ability; thus, we wondered whether teamwork was somewhat enhanced, and we also collected data on their clinical competence after our TBL class. We clarify our issues in the following parts, such as the participation of each person and the existence and acceptance of different opinions. The data are shown in Tables 3, 4. Most students choose a neutral attitude to each question. For the highest agreeable percentages, most classmates thought that TBL helped improve teamwork at the level of the acceptance of different views; on the other hand, only 24.07% of the students thought that they were concentrating when debating, which means that we should allocate more teachers to help each group to engage in the discussion section.

**Table 3:** Team work ability.

**Question**		**Frequency %**
**Most of the members of our team participated in the project discussion**		
Strong agree	16	14.81%
Agree	24	22.22%
Neutral	42	38.89%
Disagree	21	19.44%
Strong disagree	5	4.63%
**Many different opinions emerge during the team discussion**		
Strong agree	10	9.26%
Agree	31	28.70%
Neutral	47	43.52%
Disagree	16	14.81%
Strong disagree	4	3.70%
**Different views can be accepted by others in teamwork**		
Strong agree	12	11.11%
Agree	35	32.41%
Neutral	48	44.44%
Disagree	11	10.19%
Strong disagree	2	1.85%
**Every membrane has an opportunity to express their opinions in teamwork**		
Strong agree	11	10.19%
Agree	28	25.93%
Neutral	50	46.30%
Disagree	15	13.89%
Strong disagree	4	3.70%
**Everyone is focusing on the discussion in teamwork**		
Strong agree	9	8.33%
Agree	17	15.74%
Neutral	53	49.07%
Disagree	24	22.22%
Strong disagree	5	4.63%

**Table 4:** TBL on clinical ability.

**Question**		**Frequency %**
**TBL improves our clinical reasoning thinking in the problem-solving section**		
Strong agree	11	10.19%
Agree	27	25%
Neutral	54	50%
Disagree	13	12.04%
Strong disagree	3	2.78%
**TBL teaching makes us know more about eye surgery**		
Strong agree	44	40.74%
Partially agree	55	50.93%
disagree	9	8.33%
**We are more impressed with eye surgery in TBL**		
Strong agree	55	50.93%
Partially agree	45	41.67%
disagree	8	7.41%
**TBL improves our ability on how to take in and treat patients**		
Strong agree	26	24.07%
Partially agree	60	55.56%
disagree	22	20.37%

### Evaluation of students' satisfaction with TBL

**Figure 4:**
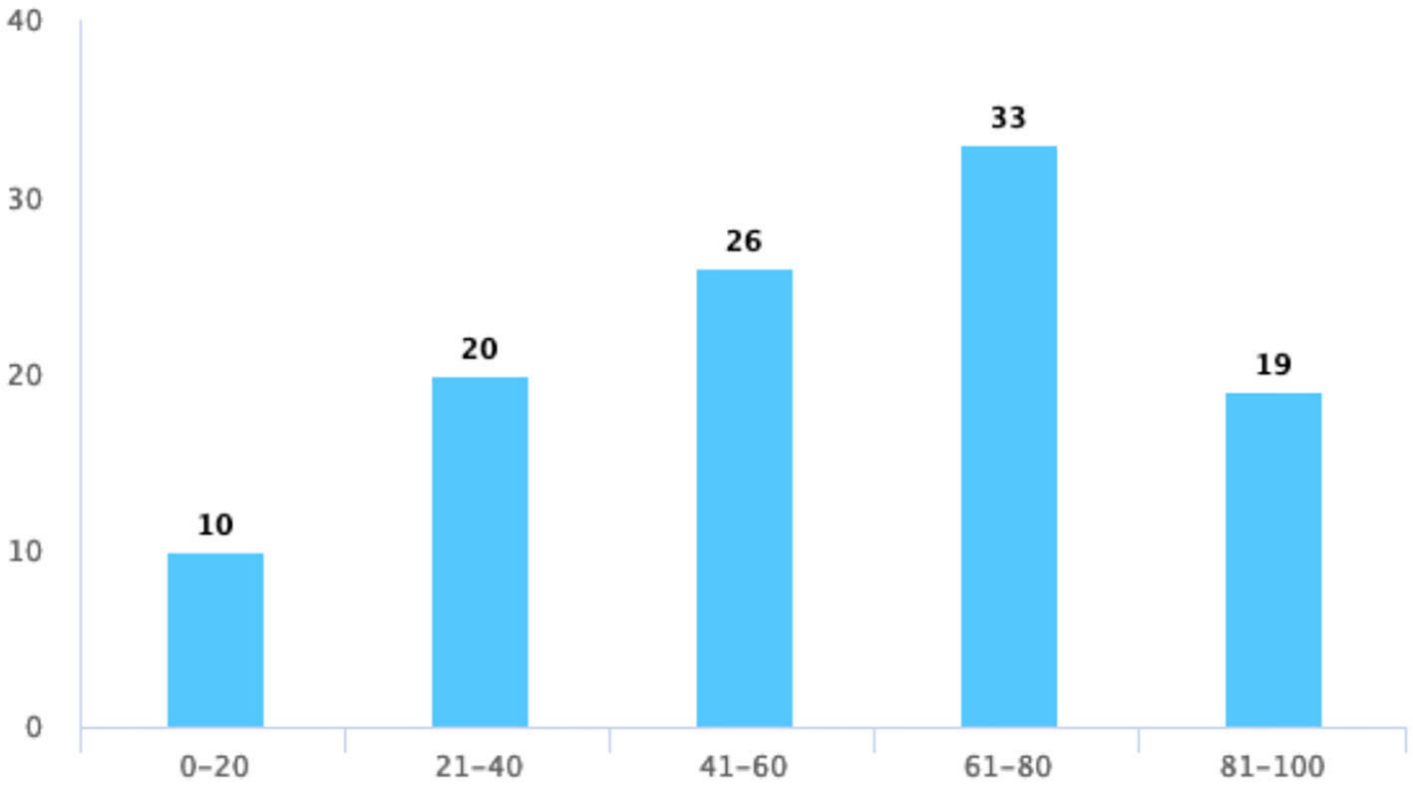
Your satisfaction with the TBL teaching evaluation.

**Figure 5:**
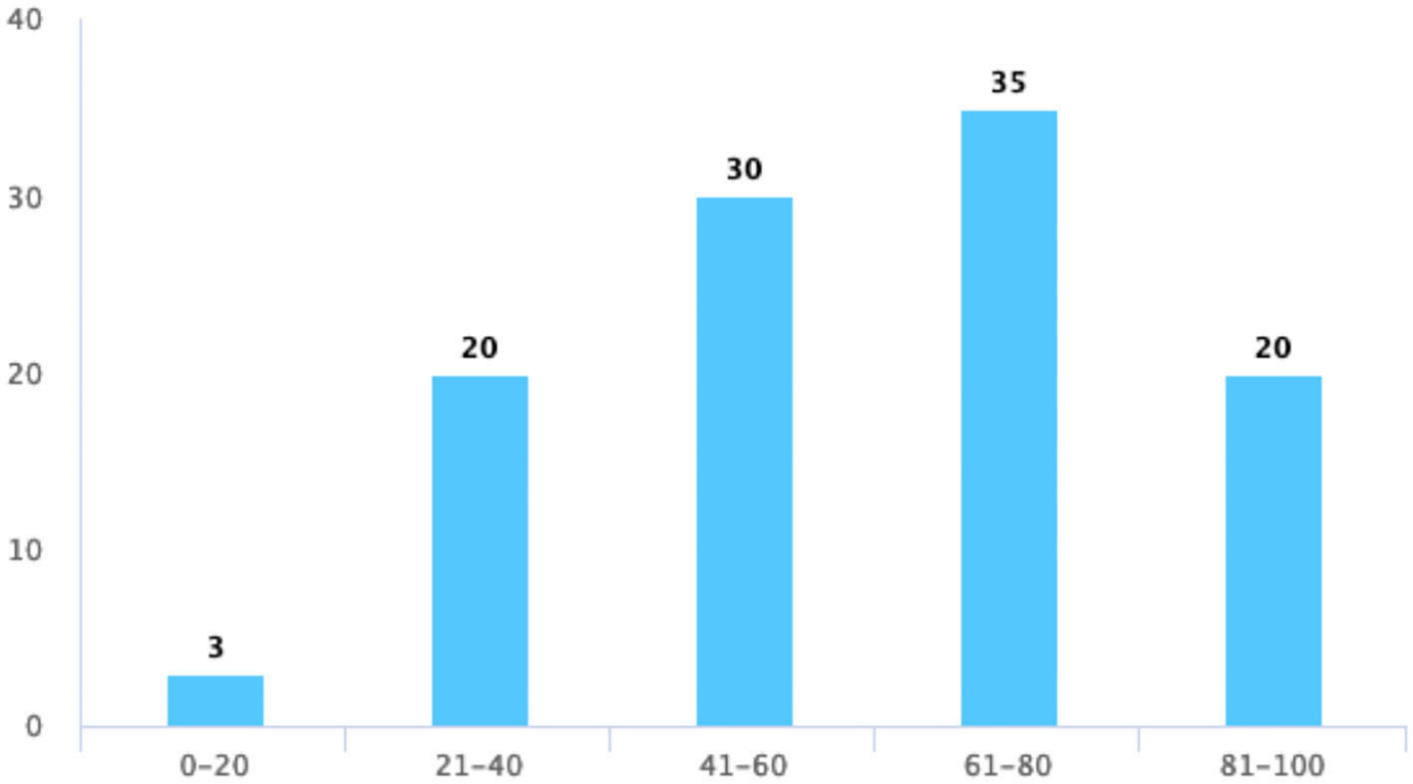
Evaluation of an active atmosphere in the TBL class.

**Figure 6:**
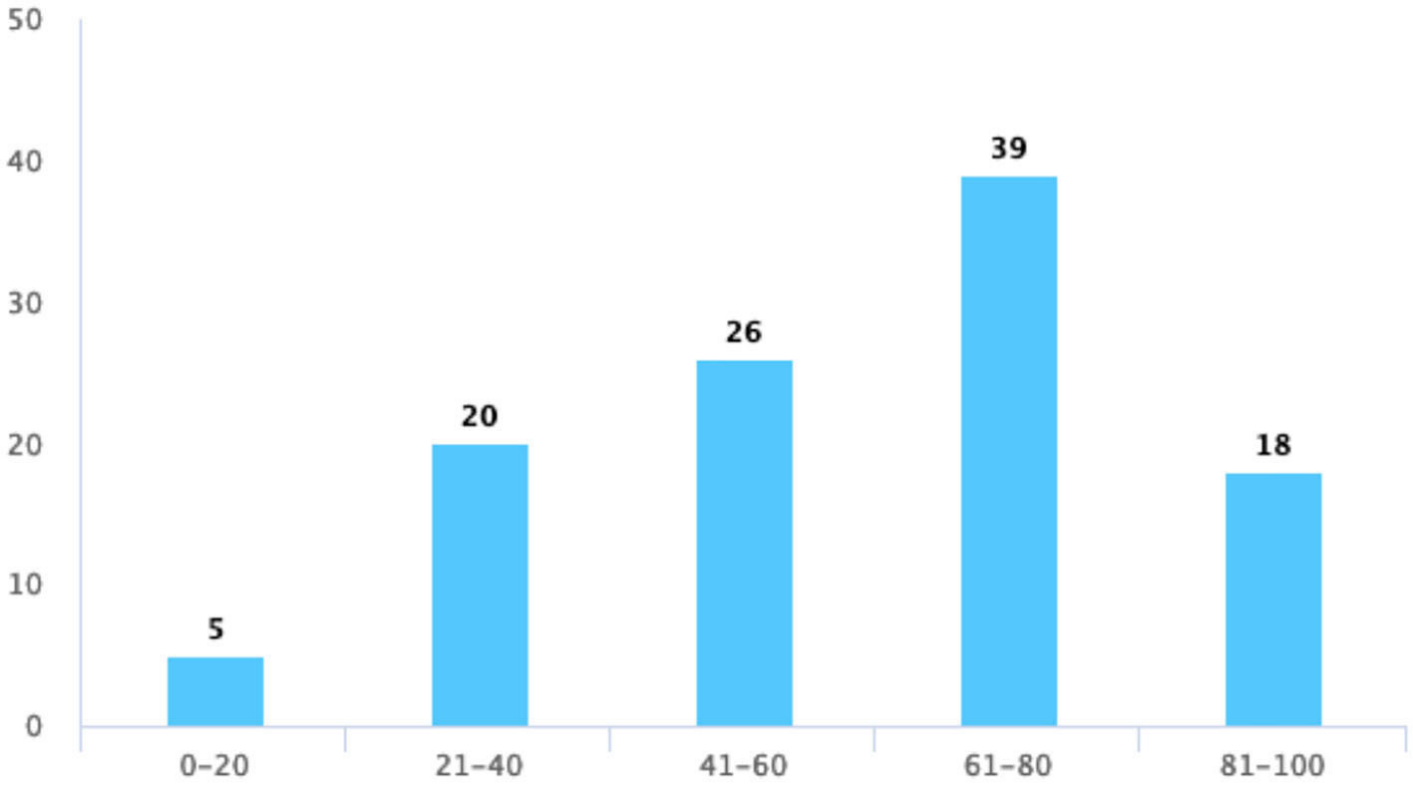
The discussion of TBL increases interactions between classmates or teachers.

**Figure 7:**
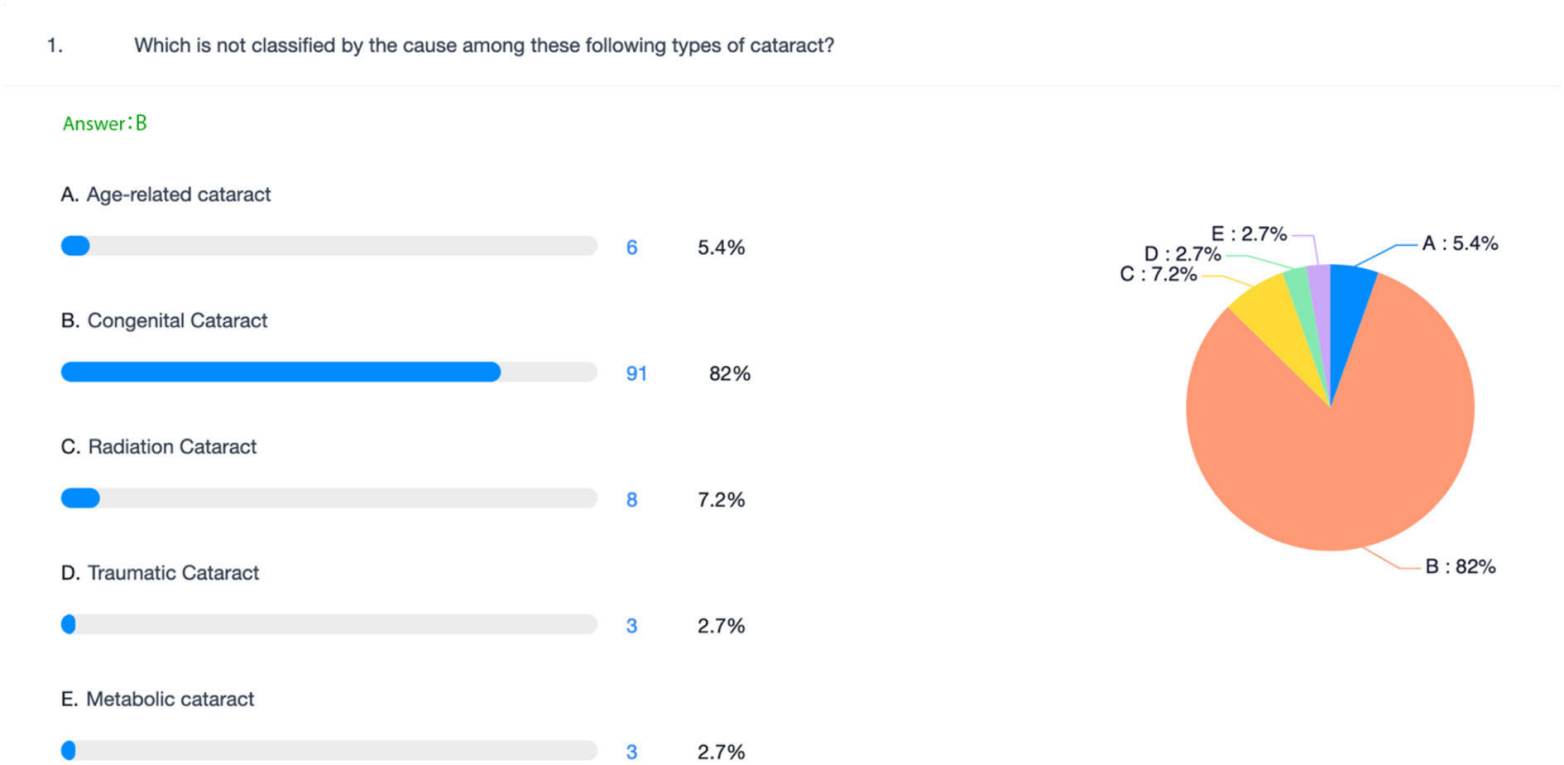
An example displays a class quiz with the Chaoxing app. Answers are shown in a pie chart with their percentages.

At last, we focused on the evaluation of students' satisfaction with TBL, where students thought that TBL helped them to master their present knowledge, provided them with more opportunities to express themselves, and had a positive impact on their learning attitudes. In Table 5, however, fewer students give their satisfaction on these three questions. Figure 4 shows an increased score of up to 80 and a slight decrease in the score from 80 to 100 points when asking about their satisfaction with TBL. The scoring trend of an active atmosphere and opportunity for discussion in the TBL class is consistent with their satisfaction with the quiz (Figures 5–7).

**Table 5:** TBL satisfaction.

**Question**		**Frequency %**
**We have no resistance to TBL**		
Strong agree	7	6.48%
Agree	13	12.04%
Neutral	49	45.37%
Disagree	32	29.63%
Strong disagree	7	6.48%
**TBL teaching mode is expected to be carried out in more subjects**		
Strong agree	7	6.48%
Agree	10	9.26%
Neutral	39	36.11%
Disagree	30	27.78%
Strong disagree	22	20.37%
**TBL teaching is an effective teaching method**		
Strong agree	6	5.56%
Agree	16	14.81%
Neutral	53	49.07%
Disagree	22	20.37%
Strong disagree	11	10.19%

## Discussion

Over the past 30 years, educators on college campuses have increasingly employed a group teaching method called TBL (16). It was stated that collaboration can play a significant role in mitigating many of the challenges facing the world of intensified competition. We embarked on a study of TBL to determine whether this instructional strategy had value for use in medical education. This study sought to explore collaborative effectiveness and medical students' perceptions of their learning experience during TBL sessions in the curriculum of ophthalmology. As TBL had a positive impact on knowledge acquisition and utilization, we choose to apply TBL to ophthalmology due to its small curriculum and independence, which is extremely applicable to analyze the role of TBL in medical education in China. Working together, the students achieved significantly higher GRAT scores than the IRAT scores. The students showed more interest and spent more time on ophthalmology. Additionally, more than half of the students were satisfied with our TBL approach.

Before our class, we used MOCC and guidebooks to publish tasks. The TBL method was unacceptable at first due to heavy tasks, and they did not have enough time to preview the entire curriculum; after several TBL training sessions, students tended to accept it and were more willing to spend time on the preview.

During our class, we used the Superstar app for a test during our TBL class, and it can show the learning results immediately so that students' wrong knowledge points are displayed in the form of a pie chart (Figure 7), which is better than the previous scratch card. It is very convenient for teachers to understand students' knowledge.

It is accepted that, with group learning methods such as TBL, groups should outperform individuals (17, 18). The analysis of pre-class tests (Figure 2) indicated that group average scores were higher than individual average scores. This result suggested that group-based learning increases problem-solving more than individuals. During group discussions, students could communicate and debate to resolve the problem. Our education seems more effective compared to the one that is acquired from listening to teachers based on the response to the questionnaire.

However, we also found that the section of group discussion was not fierce, and students were afraid to communicate with each other even if they had an idea, which might be due to the spoon-fed education pattern, and TDL is the most universal education model in China since primary school. They were afraid of expressing themselves, so they were more inclined to listen than deliver their opinions.

In our survey, we collected feedback at the beginning of our TBL, and most of the students do not accept this model. Approximately 57.8% of them are more willing to return to TDL. They complain about little time to preview due to the heavy task of their study work. In the program of medical students in China, students enter the medical undergraduate program after high school, which increases our students' need to learn more basic knowledge in their MD program and virtually increases the learning task of medical students.

Moreover, in our study, we found that TBL is superior to lecture-based learning only when students have reached a comparable understanding of ophthalmological theories; otherwise, it will be difficult for students to answer quizzes and engage in effective discussion. The data show that about 47% of them have problems with the online quizzes at the beginning of class, but at the end of several TBL classes, only 4.63% of the student agree with the difficulty of IRAT (Table 1). We thought that this would be the improvement of preview and learning ability through several rounds of training. In addition, 35% of them thought that the number of each group is overloaded, and this affects the individual discussion in GRAT and clinical problem-solving activities. A study demonstrated that the optimal size of a TBL team is considered to be five to seven members (19). In our experience, 14 members were too much for group discussion and we thought that the number to be under control should be seven as each of the members had an opportunity to discuss.

Previous studies (20) found that improving learning outcomes was one of the major benefits of TBL. We evaluated the effect of TBL on students' performance using final exam scores (FES) and analyzed the mechanisms by interpreting the questionnaire results (Tables 1–4). Our results did not show a significant improvement in FESs for TBL teaching. Although the failure rate has decreased markedly from 31 to 17, this indicates that the number of students who master the knowledge has increased. The questionnaire indicated that the enhancement of personal knowledge was improved through an interaction with team members and the amount of time spent understanding the curriculum; thus, we thought that TBL was an effective educational method for gaining knowledge and was advantageous for improving teamwork skills, independent learning skills, and knowledge application skills.

Our students are more willing to be neutral when there is a neutral choice in each quiz but are likely to partially agree if the options do not have neutral. This suggests that most of our students are neither sure about the effect of TBL on themselves nor about their future planning. However, we overlooked one significant detail that we provide answers to after the IRAT, and this may decrease their explorative learning and has also affected the group dynamics, which is essential to keep them focused. They indeed do not know what is most suitable for them. The reason for this phenomenon may be attributed to a long time of spoon-feeding education, and students are reluctant to express their radical ideas. We need to improve our discussion step since the highest proportion of disagreement on team discussion facility is understanding knowledge. At this point, TBL will be a kind of quality cultivation, learning from excellent team members.

In conclusion, we used questionnaires to gain insights into the use of TBL in medical education. Despite an increase in the use of TBL, it is not popularized in Chinese undergraduate education. We are on the way to creating a Chinese-adapted TBL model. These changes were associated with several factors at the faculty, student, course, and administrative/curricular levels. Schools that desire to implement TBL would do well to consider these characteristics when developing their implementation plans.

## Data availability statement

The raw data supporting the conclusions of this article will be made available by the authors, without undue reservation.

## Ethics statement

The studies involving human participants were reviewed and approved by the Central South University of Human Research Ethics Committee. The patients/participants provided their written informed consent to participate in this study.

## Author contributions

WW and DW designed the questionnaire. LP drew some figures. EZ distributed the questionnaire. WW analyzed the data and wrote this manuscript. XX, SX, XZ, and DW revised this manuscript. All authors contributed to the article and approved the submitted version.

## Funding

This work was supported by Hunan Province ordinary higher education reform research general education project (HNJG-2021-0311) to DW, CSU education reform research general education project (2021jy138) to DW, the key project of Postgraduate Education and Teaching Reform of CSU (2020JGA008) to DW, the key project of Academic Degree and Postgraduate Education Reform in Hunan Province (2020JGZD011) to DW. The key project of Hunan Provincial Education Department entitled Exploration and practice of classified training of clinical medical doctoral students (2020JGZX002) to XX.

## Conflict of interest

The authors declare that the research was conducted in the absence of any commercial or financial relationships that could be construed as a potential conflict of interest.

## Publisher's note

All claims expressed in this article are solely those of the authors and do not necessarily represent those of their affiliated organizations, or those of the publisher, the editors and the reviewers. Any product that may be evaluated in this article, or claim that may be made by its manufacturer, is not guaranteed or endorsed by the publisher.
